# Mitochondrial Myopathy in Follow-up of a Patient With Chronic Fatigue Syndrome

**DOI:** 10.1177/2324709615607908

**Published:** 2015-09-24

**Authors:** Fernando Galán, Isabel de Lavera, David Cotán, José A. Sánchez-Alcázar

**Affiliations:** 1 University of Seville, Seville, Spain; 2 Universidad Pablo de Olavide-Consejo Superior de Investigaciones Científicas-Junta de Andalucía, Seville, Spain

**Keywords:** chronic fatigue syndrome, myalgic encephalomyelitis, mitochondrial myopathy, occipital neuralgia, riboflavin therapy

## Abstract

*Introduction*. Symptoms of mitochondrial diseases and chronic fatigue syndrome (CFS) frequently overlap and can easily be mistaken. *Methods*. We report the case of a patient diagnosed with CFS and during follow-up was finally diagnosed with mitochondrial myopathy by histochemical study of muscle biopsy, spectrophotometric analysis of the complexes of the mitochondrial respiratory chain, and genetic studies. *Results*. The results revealed 3% fiber-ragged blue and a severe deficiency of complexes I and IV and several mtDNA variants. Mother, sisters, and nephews showed similar symptoms, which strongly suggests a possible maternal inheritance. The patient and his family responded to treatment with high doses of riboflavin and thiamine with a remarkable and sustained fatigue and muscle symptoms improvement. *Conclusions*. This case illustrates that initial symptoms of mitochondrial disease in adults can easily be mistaken with CFS, and in these patients a regular reassessment and monitoring of symptoms is recommended to reconfirm or change the diagnosis.

## Introduction

Chronic fatigue syndrome (CFS)—also known as myalgic encephalomyelitis (ME)—is characterized by profound fatigue that is not improved by bed rest and that may be worsened by physical or mental activity. Symptoms affect several body systems and may include muscle pain, disturbed sleep, and cognitive dysfunction among others.^1^ Its diagnosis is nonspecific and symptom based, and no laboratory tests can be used to diagnose CFS. Research over the years has identified many underlying biological abnormalities, mitochondrial dysfunction among them, but the etiology remains uncertain.^2^ No intervention has been proven effective in restoring the ability to work.^3^

Primary mitochondrial diseases are those caused by mutations in mitochondrial DNA and/or nuclear DNA genes that impair mitochondrial respiratory chain function. Mitochondrial disease in adults is often presented in a subtle way, with subjective symptoms such as fatigue and weakness that often tend to develop over the years and is rarely diagnosed when symptoms are just beginning. Many patients are oligosymptomatic, or have overlapping, poorly defined phenotype in the presentation, and thus clinicians must have a high index of suspicion when considering the possibility of mitochondrial disease.^4,5^ Symptoms may show wide variability, from relatively nonspecific exercise intolerance or painful muscle cramps caused by exercise to muscle weakness in a predominantly proximal distribution. Despite the subjective weakness, many patients after a careful physical examination early on can reveal very slight weakness.^6^ Currently, diagnosis relies on family and personal history, physical examination, histological and immunohistochemical studies, and enzymatic analysis of the complexes of the respiratory chain in muscle biopsy samples, and when available, genetic analysis of nuclear and mitochondrial DNA.

The symptoms of mitochondrial diseases and CFS overlap—such as fatigue, muscle pain, and abdominal pain, among other—and can easily be mistaken. To the best of our knowledge, no case has been reported in any patient with CFS that was finally diagnosed with mitochondrial disease.

There are currently no known treatments to halt disease progression. Recently, Parikh et al stated,

We agree that therapies using vitamins and cofactors have value, though there is debate about the choice of these agents and the doses prescribed. Despite the paucity of high-quality scientific evidence these therapies are relatively harmless and may alleviate select clinical symptoms.^7(p414)^

This led us to report the case of a patient who was diagnosed with CFS and during follow-up the final diagnosis was of mitochondrial myopathy. Treatment with large doses of riboflavin and thiamine resulted in a striking and sustained improvement.

## Case Report

A 30-year-old Caucasian male, philosophy graduate, came to our Department of Internal Medicine, referring a history of prolonged fatigue for more than 10 consecutive months, being more evident as the day progressed, and did not improve with bed rest and worsened with physical and mental effort, significantly interfering with daily activities and work. He also reported that was accompanied by unrefreshing sleep, impairment in short-term memory and concentration, together with headache behind the eyes and in the back of the head and postexertional malaise that lasted more than 24 hours.

These symptoms began approximately 6 months after the diagnosis of infective endocarditis of the native valve with negative blood cultures, treated successfully with antibiotics, replacement of the aortic valve, and oral anticoagulation.

The patient also complained of other medical conditions, such as dry eye sensation, slow digestion with postprandial fullness, and Raynaud’s phenomenon in the coldest winter months. He knew it from his youth but could not specify the age of onset.

Family history included the following: mother with mild hypertension; angina pectoris, hypertension, and dyslipidemia in the father; 2 sisters and 2 nephews apparently healthy.

Physical examination revealed only a systolic murmur II/VI from the prosthetic valve in aortic area and no other abnormalities.

Routine hematological and biochemical parameters in blood and urine showed no abnormalities, except international normalized ratio 2.8. Other tests performed were iron homeostasis, basal and 2-hour plasma glucose test, thyroid function, creatine kinase, basal cortisol, 25(OH)D, folic acid, B_12_, B_6_ serum level, cell-mediated and humoral immunity, C-reactive protein, angiotensin-converting enzyme levels, serum protein electrophoresis, antinuclear antibody (ANA), anti-Ro (SSA) and anti-La (SSB), anti-endomysial, anti-neutrophil cytoplasmic antibodies, as well as cryoglobulins, and all were negative. Serologic tests for *Borrelia burgdorferi, Brucella, Chlamydia pneumoniae, Bartonella henselae* and *B quintana, Coxiella burnetii, Helicobacter*, HBsAg, anti–hepatitis C virus, anti-cytomegalovirus IgG and IgM antibodies, and Epstein–Barr virus (EB V) IgM antibodies were negative. Only EBV IgG was positive.

Doppler echocardiographic evaluation of the function of the aortic valve prosthesis and the heart was normal, as well as electrocardiogram. Tear breakup time and Schirmer test were equivocal. Upper gastrointestinal endoscopy was normal.

Therefore, the patient appeared to meet the CDC-1994/Fukuda criteria for CFS,^1^ and the diagnosis of CFS was made. He was treated for 1 year with cognitive behavioral therapy, graded exercise therapy, and antidepressants, finding very slight improvement, and the headache did not respond to triptans or ergot alkaloids and responded only partially to nonsteroidal anti-inflammatory drugs.

As recommended, patients with ME/CFS require a regular reassessment and follow-up of symptoms (annually) to reconfirm or change the diagnosis. Therefore, we followed the patient for several years paying attention to the emergence of new symptoms and signs.

During follow-up, at the end of the second year, the patient reported feeling more tired, lack of energy, exhausted, with heaviness in the arms and legs, muscle pain, and proximal muscle weakness became more evident throughout the day, and even more after exercise often accompanied by muscle cramps. About 6 months later, the patient began to feel tingling in the feet, and then in the hands and sometimes burning sensation, along with a sense of restless legs. In the middle of the third year, these symptoms were more evident and the patient also complained of dry eyes, dry mouth, orthostatic intolerance, intestinal motility disorder with very annoying abdominal bloating, and with persistence of slow digestion with postprandial fullness.

The headache was now more accurately described as unilateral paroxysmal pain in the back of the head characterized by the sensation of pain as stabbing or electric shock that radiates to the area of the occipital and parietal scalp, and sometimes on the front and periorbital area. When the pain was strong, it was associated with pain behind the eye on the affected side. The pain was reproduced locally by manually compressing the greater occipital nerve. Given the characteristics of this headache, with a frequency of about once every 2 weeks, our diagnosis was occipital neuralgia. Furthermore, triptans and ergot alkaloids therapies were unsuccessful.

On examination, the patient weighed 75 kg and measured 168 cm of height. Body mass index was 26.6 kg/m^2^. At rest the blood pressure was 115/70 mm Hg, the pulse 83 beats per minute, and the respiratory rate 18 breaths per minute. Systolic murmur of aortic prosthetic valve had not changed. Arterial pulses were normal. Muscle strength in the arms was 4/5 proximal, 4−/5 proximal legs, and distal 5/5 both (according to MRC scale). Sensory examination showed a slight decrease in sensitivity to light touch and pinprick symmetric distribution within a distal-to-proximal gradient in the upper and lower extremities. Deep tendon reflexes, vibration, and proprioception were preserved. Romberg sign was absent, and the gait was normal.

The laboratory tests were repeated, and the results were unchanged.

Head-up tilt-table testing was positive for postural hypotension. Cranial and cervical spine magnetic resonance imaging and thorax and abdominal computed tomography scans were normal. Electromyography and nerve conduction studies were carried out in the upper and lower extremities and were normal. Tear breakup time and Schirmer test were positive.

After these results, we considered the possibility of mitochondrial myopathy in this patient. After obtaining informed consent, a histochemical analysis of a deltoid muscle biopsy revealed 3% ragged-blue fibers with the succinate dehydrogenase (SDH) stain.

In addition, a lip biopsy was also performed and was normal, dismissing Sjögren’s syndrome.

Spectrophotometric enzyme analysis^8^ of a fresh muscle extract showed a severe deficiency of activity in complex I (nicotinamide adenine dinucleotide: ubiquinone oxidoreductase) and IV (cytochrome c oxidase) below 42% and 70% of the minimum reference of control value normalized to citrate synthase activity, respectively. Thus, he was diagnosed of definite respiratory chain disorder because of fulfillment of 2 major criteria.^9^ All these findings were consistent with a primary mitochondrial myopathy.

Mitochondrial genome sequencing in blood sample was performed using the Sanger method, which revealed several mtDNA variants (A750G, T1189C, and A1438G) (Sistemas Genómicos, Valencia, Spain).

Therefore, the final diagnosis was adult-onset mitochondrial myopathy, with clinical manifestation of peripheral sensory neuropathy, autonomic symptoms, and occipital neuralgia.

**Figure 1.**
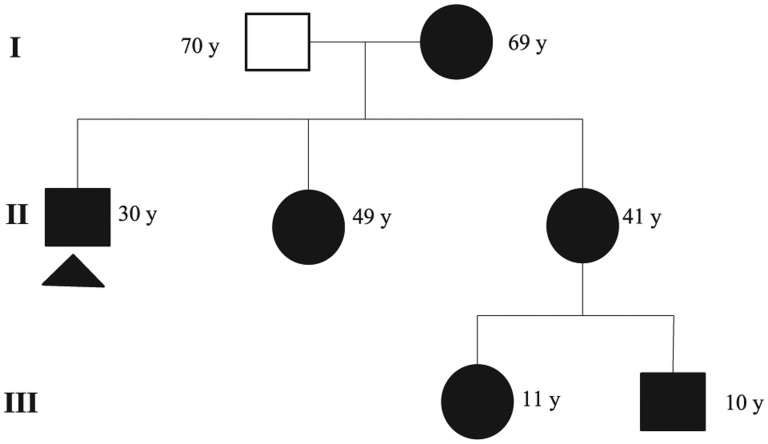
Diagram of the family pedigree. Solid symbols indicate clinically affected individuals, and open symbols unaffected individuals. Age in years is shown beside of subject symbols. Arrowhead pointing to the proband.

Subsequently, we conducted a clinical study of the family, and the results are shown in Table 1. We can observe that all adults have muscle symptoms, sensory peripheral neuropathy, restless legs, occipital neuralgia, and autonomic manifestations—dry eyes, mouth, Raynaud phenomenon, postural hypotension, gastrointestinal dysmotility. Figure 1 shows the diagram of the family pedigree.

**Table 1.** Clinical Findings of the Patient’s Family Members in This Study.

	Relatives and Age (Years)					
Symptoms	Proband, 30 Years	Mother, 69 Years	1st Sister, 49 Years	2nd Sister, 41 Years	Daughter of 2nd Sister, 11 Years	Son of 2nd Sister, 10 Years
Fatigability	+ + +	+ +	+ +	+ + +	+ +	+
Exercise intolerance	+ + +	+ +	+ +	+ + +	+ +	+
Muscle weakness	+ +	+ +	+ +	+ +	+ +	+
Myalgia	+ + +	+ +	+ +	+ + +	+ +	+
Muscle cramps	+ +	+	+	+ +	+	+
Impaired memory and concentration	+ + +	+	+	+ +	−	−
Peripheral SNP	+ + +	+ +	+ +	+ +	+	+
Restless legs	+ + +	+ + +	+ +	+ + +	+ +	+ +
Occipital neuralgia	+ + +	+ +	+ +	+ + +	+	+
Dry eyes and mouth	+ + +	+ + +	+ +	+ + +	+	+
Raynaud phenomenon	+ + +	+ + +	+ + +	+ + +	+ +	+ +
Postural hypotension	+ + +	+	+ +	+ +	+	+
Gastrointestinal dysmotility	+ + +	+ + +	+ + +	+ + +	−	−

Abbreviations: + + + = marked; + + = moderate; + = mild; − = none.

In our patient, treatment started on a regimen of a combination of riboflavin (100 mg 3 times per day) and thiamine (300 mg/day). During the first month of treatment, the patient started a marked and sustained improvement, which continues (5 years later) in the tiredness, impairment in short-term memory and concentration, muscle weakness, cramping in muscles after exercise, and the patient can now perform exercise, such as bicycling.

However, peripheral sensory neuropathy improved slightly with the vitamin regimen, but a manifest and sustained improvement was achieved together with the occipital neuralgia, when added pregabalin therapy at doses of 50 mg at night. Replacement therapy with tears and saliva provided a clear and sustained improvement of dry eyes and dry mouth.

## Discussion

In clinical practice, it is not uncommon to find patients with overlapping symptoms of some common conditions, including, among others, CFS, fibromyalgia, depression, and psychosomatic illness. And more recently may also include mitochondrial disease, although less common but not very rare. In fact, a recent general population study reported a prevalence in carriers of mtDNA mutations of about 1 in 200 persons^9^; however, this study was limited to testing only the common mutations, underestimating the true prevalence of all types of mitochondrial disease.

And even more recently, Gorman et al reported “the total prevalence of adult mitochondrial disease in the North East of England, including pathogenic mutations of both the mitochondrial and nuclear genomes (1 in 4,300), is among the commonest adult forms of inherited neurological disorders.” And the nuclear mutations account for approximately one third of the prevalence for mtDNA mutations.^10^

The mitochondrial disease may be hard to diagnose and could be partly due to the lack of awareness by the physician and the general population, especially in the initial presentation in adults, due to the extreme clinical variability among patients, intermittent and variable symptoms within individual patients, and the lack of sensitivity in noninvasive diagnostic tests.

This is the case of our patient who initially had symptoms compatible with a diagnosis of CFS, but during follow-up for several years was diagnosed with adult-onset mitochondrial myopathy with clinical manifestation of peripheral sensory neuropathy, autonomic symptoms, and occipital neuralgia, due to the appearance of new symptoms and signs.

The alert symptoms of mitochondrial myopathy appeared at the end of the second year. Muscle tissue is one of the tissues of the body with the highest energy demands, and therefore often myopathy is the first symptom in adults. The patient had minimal objective findings, and a careful physical examination revealed mild proximal muscle weakness, manifested more in the legs. The muscle studies demonstrated a definite mitochondrial respiratory chain disorder.^11^

Mitochondrial DNA variants (A750G, T1189C, and A1438G) found in this patient have been reported in patients with idiopathic sensorineural hearing loss; however, the pathogenicity of these variants should be established, as well as the appearance in people with normal hearing, in order to define its possible correlation with NSHL additional and/or aminoglycoside-induced HL.^12^

This contributed to a more complete and thorough clinical study of the whole family, being able to observe—to a greater or lesser extent—similar symptoms, which strongly suggests a possible maternal inheritance (Table 1 and Figure 1). Family history may be overlooked due to mild or oligosymptomatic disease, variability in clinical manifestations, or because many relatives may be asymptomatic carriers.

Sensory peripheral neuropathy in our patient was manifested clinically as small fiber neuropathy with sensory symptoms and autonomic symptoms. Chronic axonal sensorimotor polyneuropathy is the most common pattern, but small fiber neuropathy^13^ and autonomic nervous system dysfunction^14^ have also been reported. This makes sense from a pathophysiological point of view, because small fiber neuropathy affects small somatic fibers and autonomic fibers, being able to give sensory and autonomic symptoms. In addition, electromyography and nerve conduction studies often are normal in pure small fiber neuropathies.

Idiopathic occipital neuralgia is a form of neuropathic pain and there has been no reports in patients with mitochondrial disease. Occipital neuralgia can be difficult to differentiate from migraine, but in our case—based on careful history and physical exam—was not better accounted by an alternate diagnosis. These patients sometimes report front and periorbital pain radiation through trigeminocervical interneural connections in the spinal trigeminal nucleus.^15^

Interestingly, treatment with high doses of riboflavin and thiamine was notably effective in reducing the clinical symptoms. The outcome in our patient and their relatives was a striking and sustained improvement of tiredness, muscle weakness, and cramping in muscles after exercise. This has been already reported in some patients with mitochondrial disease both in vivo^16^ and in vitro.^17^

The question is whether the patient has had 2 consecutive diseases or just a misdiagnosed disease at initial presentation. Our first thought was that we could not deny the possibility of 2 clinical diseases, related or not from a physiological point of view. Mitochondrial dysfunction in CFS is an open debate with conflicting results. Recent studies^2,18^ reported deficiencies in the provision of ATP in neutrophils of patients with CFS due to lack of essential substrates and partial blocking of the translocator protein sites in mitochondria. However, the authors cannot assert that the mitochondria in other cells are dysfunctional at the same degree. But another recent study^19^ reported that ATP production rate—in cells from skeletal muscle biopsies—was within the normal range in all patients with CFS while it was decreased in patients with mitochondrial disorders. And they conclude saying that mitochondrial function is not affected in skeletal muscle of patients with CFS.

Although common sense prevailed and our final thought was that from the beginning the patient could be a carrier of mitochondrial myopathy and the warning signs appeared over the following years. This allowed us to make the diagnosis of mitochondrial myopathy in adulthood, and with the onset of other symptoms could be diagnosed as nonsyndromic mitochondrial disease with possible maternal transmission. Adults with mitochondrial disease commonly present with clinical manifestations that do not conform to any of the classic syndromes, also known as nonsyndromic mitochondrial disease.^20^

In conclusion, this case can be useful to illustrate that initial symptoms of mitochondrial diseases in adults can easily be mistaken with CFS and it is recommended that these patients have a regular reassessment and follow-up (annually) of symptoms to reconfirm or change the diagnosis. In addition, large doses of riboflavin and thiamin are also suggested as a treatment option, which can alleviate some of the clinical symptoms in adults.
